# Small Airway Dysfunction in Chronic Bronchitis with Preserved Pulmonary Function

**DOI:** 10.1155/2022/4201786

**Published:** 2022-08-24

**Authors:** Qi Ding, Bai-Bing Mi, Xia Wei, Jie Li, Jiu-Yun Mi, Jing-Ting Ren, Rui-Li Li

**Affiliations:** ^1^Department of Pulmonary and Critical Care Medicine, The Ninth Hospital of Xi'an Affiliated with Xi'an Jiaotong University, Xi'an, Shaanxi 710054, China; ^2^Department of Epidemiology and Biostatistics School of Public Health, Xi'an Jiaotong University Health Science Center, Xi'an, Shaanxi 710061, China

## Abstract

Impairment of pulmonary function was evaluated in chronic bronchitis patients with preserved ratio impaired spirometry (PRISm). We retrospectively collected clinical data from 157 chronic bronchitis (CB) and 186 chronic obstructive pulmonary disease (COPD) patients between October 2014 and September 2017. These patients were assigned to three groups: control (normal pulmonary function), PRISm (forced expiratory volume in 1 second [FEV1]/forced vital capacity [FVC] ≥ 0.7, FEV1 < 80% of predicted value), and COPD (FEV1/FVC <0.7) groups. Because small airway function was the main focus, in the COPD group, only patients in accordance with the Global Initiative for Chronic Obstructive Lung Disease (GOLD) grades 1 and 2 were included. Evaluation of pulmonary function (including impulse oscillometry) was performed and compared among these groups. Compared with the control group, the PRISm and COPD groups showed statistically significant differences in the predicted FEV1% (*p* < 0.001), maximal expiratory flow (MEF) 25% (*p* < 0.001), MEF50% (*p* < 0.001), maximal midexpiratory flow (MMEF) 25–75% (*p* < 0.001), residual volume (RV)/total lung capacity (TLC; *p* < 0.001), FVC% (*p* < 0.001), total respiratory resistance and proximal respiratory resistance (R5-R20; *p* < 0.001), respiratory system reactance at 5 Hz (X5; *p* < 0.001), resonant frequency (Fres; *p* < 0.001), and area of reactance (Ax; *p* < 0.001). However, the predicted FEV1% and RV/TLC were similar between the PRISm and COPD groups (*p*=0.992 and 0.122, respectively). PRISm is a nonspecific pattern of pulmonary function that indicates small airway dysfunction and may increase the risk of transformation to obstructive ventilation dysfunction. This trial is registered with ChiCTR-OCH-14004904.

## 1. Introduction

Chronic obstructive pulmonary disease (COPD) is characterized by persistent respiratory symptoms and progressive airflow limitation [1]. It is closely related to chronic bronchitis (CB) and emphysema. Therefore, CB often has been mistakenly considered as a subgroup of COPD. Acute exacerbations of COPD are considered to be exacerbations of respiratory symptoms that require additional therapy. However, some CB patients with respiratory symptoms present with a reduced forced expiratory volume in 1 second (FEV1) and normal FEV1/forced vital capacity (FVC). This nonspecific type of pulmonary dysfunction was first reported at the beginning of the twenty-first century [2] and was defined as preserved ratio impaired spirometry (PRISm) in recent years (FEV1/FVC ≥ 0.7, FEV1 < 80% of predicted value) [3]. PRISm is associated with increased respiratory symptoms, exacerbations, and mortality [4, 5]. It is not a specific lung disease, but an unstable state of lung function. Subjects with PRISm usually present with heterogeneous lung function impairment [4, 6] and can transit to other lung function categories with normal or obstructive spirometry [7]. Recently, it has been confirmed that even smokers with PRISm, whose symptoms do not meet the definition of COPD, can experience airway impairment and exacerbations [8, 9]. However, these alterations are irrelevant to the acceleration of pulmonary function deterioration [10].

Impairment of small airway function and respiratory symptoms are common in patients without COPD [11]. Traditional airway resistance parameters, such as FEV1, FEV1/FVC, the predicted maximal expiratory flow (MEF) 25%, MEF 50%, and maximal midexpiratory flow (MMEF) 25–75%, are often used to measure small airway function, but their value remains limited [12]. The impulse oscillometry system (IOS) is a noninvasive method, first described in 1956 [13], to measure respiratory system resistance and reactance during normal breathing [14]. It is easy to be performed in patients who are unable to undergo spirometry [15]. The IOS indices of total respiratory resistance and proximal respiratory resistance (*R*5–*R*20) and respiratory system reactance at 5 Hz (*X*5) can provide important information regarding distal/small airways. However, the area of reactance (Ax) is closely related to *R*5–*R*20, which reflects peripheral airway function [16]. Investigators presumed that the IOS may be more sensitive in the diagnosis of peripheral airway disease [17].

To our knowledge, few studies have been performed regarding the impact of PRISm on small airways. Considering that the FEV1/FVC ratio is not sensitive enough to diagnose early airway disease, our study focused on the influence of pulmonary function (especially small airway function) in CB patients with PRISm, with or without a smoking history.

## 2. Materials and Methods

### 2.1. Study Design and Patients

This was a retrospective, observational, cross-sectional study with consecutive patients who were admitted to our Department of Pulmonary and Critical Care Medicine. All enrolled subjects completed pulmonary function tests on the same day and their data were analyzed. Patient baseline characteristics (including age, sex, body mass index, smoking history, blood test, and COPD Assessment Test score) were collected.

Patients who had been hospitalized for an acute exacerbation of CB or COPD at the Ninth Hospital of Xi'an affiliated to Xi'an Jiaotong University (Xi'an, China) between October 2014 and September 2017 were included in the study. The diagnostic criterion for CB was the presence of chronic cough and sputum for at least 3 months in a year, persisting for more than 2 consecutive years [18]. The diagnostic criterion for COPD was a FEV1/FVC <70% after bronchodilator use. We excluded subjects who (1) were <40 years old; (2) were pregnant; (3) exhibited serious comorbidities of the respiratory system, such as a tumor, pulmonary embolism, interstitial lung disease, and/or active tuberculosis; (4) previously underwent a surgical treatment; or (5) exhibited combined asthma, severe heart, liver, or kidney dysfunction. According to the results of the pulmonary function assessment, patients were divided into control (FEV1/FVC ≥ 0.7 and predicted FEV1 ≥ 80%; *n* = 77), PRISm (FEV1/FVC ≥ 0.7 and FEV1 < 80% of predicted value; *n* = 80), and COPD groups (FEV1/FVC <0.7; *n* = 186). Airflow limitation severity in COPD was further classified into grades 1 through 4 of the Global Initiative for Chronic Obstructive Lung Disease (GOLD). Only GOLD grades 1 and 2 were enrolled. Because of the smaller sample sizes, GOLD grades 1 and 2 were combined.

This study was approved by the Chinese Clinical Research Registry (No. ChiCTR-OCH-14004904). Written informed consent was obtained from each patient.

### 2.2. Measurements

#### 2.2.1. Pulmonary Function Tests

All patients underwent spirometry (Jaeger MasterScreen, CareFusion Germany 234 GmbH, Germany) after using an inhaled bronchodilator (200 *μ*g salbutamol). The predicted FEV1%, FEV1/FVC, FEV1/FEV6, FVC%, MEF25%, MEF50%, MMEF25–75%, and residual volume (RV)/total lung capacity (TLC) were measured by spirometry. The IOS parameters were as follows: respiratory resistance at 5 (*R*5) and 20 Hz (*R*20), *R*5–*R*20, *X*5, resonant frequency (Fres), and Ax. The procedure was performed following the American Thoracic Society and European Respiratory Society recommendations [19, 20].

## 3. Statistical Analysis

SPSS 19.0 software (IBM Corporation, Armonk, NY, USA) was used to statistically analyze the data. Continuous variables with a normal distribution were presented as mean ± standard deviation (SD), whereas categorical variables were compared using a chi-squared test. One-way analysis of variance (ANOVA) and least significant difference comparisons were performed, if appropriate. Spearman correlation was used to assess the relationship between pulmonary function and IOS measurements. *P* values were two-sided, and a *P* < 0.05 was defined as a statistically significant difference.

## 4. Results

### 4.1. Patient Characteristics

**Figure 1:**
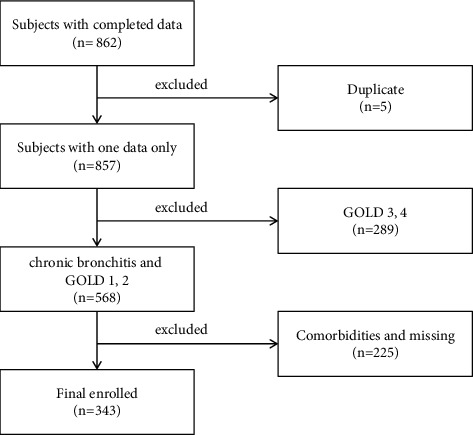
Patient selection. Totally, 862 patients were enrolled. 5 patients who rehospitalization during 2014–2017; 289 patients who defined as GOLD grades 3 and 4; and 225 patients who exhibited comorbidities and/or data missing were excluded. Complete data were available for 157 CB and 186 COPD patients.

Complete pulmonary function data were available for 157 CB and 186 COPD patients. The flow chart of subject screening is shown in Figure 1. A total of 862 patients were initially enrolled. Patients who were rehospitalized (*n* = 5), those who were defined as GOLD grades 3 and 4 (*n* = 289), and those who exhibited comorbidities and/or data missing (*n* = 225) were excluded. Finally, a total of 343 patients were included in this study, including 226 smokers, 117 nonsmokers, and 100 ex-smokers who quit smoking for more than 1 year. The baseline characteristics of the three groups are shown in Table 1. No significant differences were observed in age, body mass index, and eosinophil count among these three groups.

## 5. Comparison of Pulmonary Function Measurements (Including IOS)

There were statistically significant differences in the predicted FEV1% (*p* < 0.001), FVC% (*p* < 0.001), MEF25% (*p* < 0.001), MEF50% (*p* < 0.001), MMEF25–75% (*p* < 0.001), RV/TLC (*p* < 0.001), *R*5–*R*20 (*p* < 0.001), *X*5 (*p* < 0.001), Fres (*p* < 0.001), and Ax (*p* < 0.001) in the PRISm and COPD groups, compared with the control group. Parameters indicative of reactive airway resistance decreased, including the predicted FEV1%, MEF25%, MEF50%, and MMEF25–75%. By contrast, the air trapping (RV/TLC) and absolute values of respiratory system impedance parameters (*R*5–*R*20, *X*5, Fres, and Ax) increased. The predicted FVC% (*p* < 0.01), FEV1/FVC (%) (*p* < 0.01), FEV1/FEV6 (%) (*p* < 0.01), MEF25% (*p* < 0.01), MEF50% (*p* < 0.01), MMEF 25–75% (*p* < 0.01), *R*5–*R*20 (*p* < 0.01), X5 (*p* < 0.05), Fres (*p* < 0.05), and Ax (*p* < 0.01) in PRISm group were significantly different compared with those in the COPD group. However, the levels of the predicted FEV1% and RV/TLC were not statistically different between the PRISm and COPD groups (*p*=0.992 and 0.122, respectively; Table 2).

Furthermore, when we stratified patients in the COPD group into GOLD grades 1 (*n* = 22) and 2 (*n* = 164) subgroups, there were no statistically significant differences in the predicted MEF25% (*p*=0.25), MEF50% (*p*=0.319), and MMEF25–75% (*p*=0.314) between the PRISm and GOLD grade 1 groups; however, both groups were significantly different compared with the control group (*p*=0.005). The IOS indices of *R*5–*R*20 (*p*=0.238), *X*5 (*p*=0.089), and Ax (*p*=0.342) showed no significant differences between the PRISm and GOLD grade 1 groups.

### 5.1. Relationships between IOS and Other Pulmonary Function Measurements

**Figure 2:**
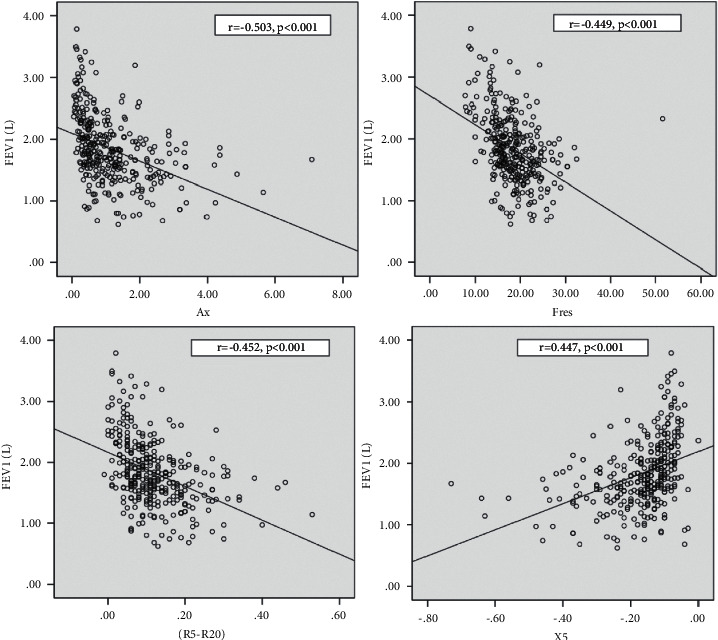
Relationship between FEV1 and IOS parameters. Statistical analyses are performed with Spearman correlation to compare correlation between FEV1 and IOS parameters. Moderate negative correlations were observed in FEV1 and Ax (*r* = −0.503), Fres (*r* = −0.449), and *R*5–*R*20 (*r* = −0.452); *p* < 0.001. By contrast, moderate positively related to FEV1 and X5 (*r* = 0.477); *p* < 0.001.

**Figure 3:**
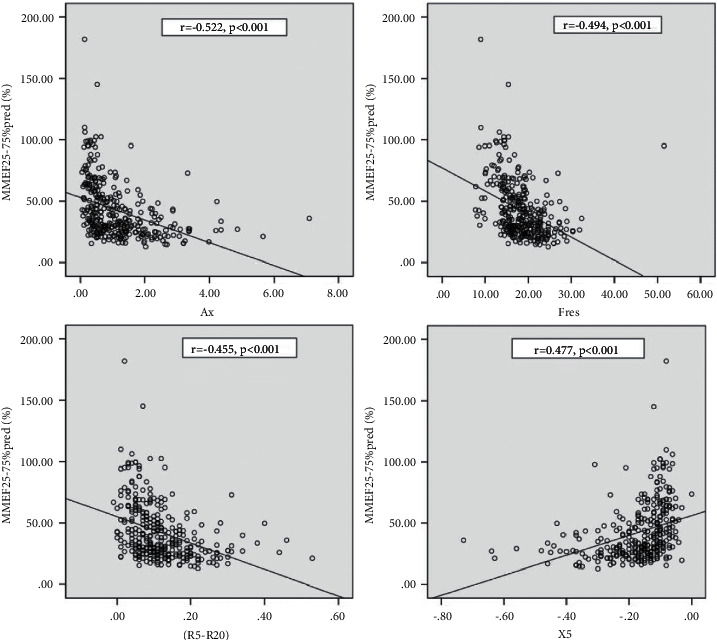
Relationship between MMEF 25–75% pred and IOS parameters. Statistical analyses are performed with Spearman correlation to compare correlation between MMEF 25–75% pred and IOS parameters. Significant negative correlations were observed in MMEF 25–75% pred and Ax (*r* = −0.522), Fres (*r* = −0.494), and *R*5–*R*20 (*r* = −0.455); *p* < 0.001. By contrast, moderate positively related to MMEF 25–75% pred and *X*5 (*r* = 0.477); *p* < 0.001.

Moderate negative correlations were observed among *R*5–*R*20, Ax, Fres, and FEV1 (*r* = −0.452, −0.503, and −0.449, respectively; *p* < 0.001). In addition, we found significant negative correlations among *R*5–*R*20, Ax, Fres, and the predicted MMEF25–75% (*r* = −0.455, −0.522, and −0.494, respectively; *p* < 0.001). *X*5 was moderately positively related to the predicted FEV1% and MMEF25–75% (*r* = 0.477; *p* < 0.001; Figure 2 and 3).

## 6. Discussion

Although progress in the treatment and prevention of acute exacerbations has been made in recent years, few advances have been made to prevent COPD progression and reduce the mortality [21]. Part of the reason might because patients generally go to the hospital at late stage of the disease, and their lung function impairment is difficult to reverse. Therefore, early diagnosis and timely clinical intervention for high-risk groups with no obvious symptoms are important to reduce the disease burden of COPD. PRISm is associated with increased symptoms, radiographic emphysema and gas trapping, exacerbations, and progression to COPD [22]. However, little is known about the pathophysiological mechanisms of the progression from PRISm to COPD.

The prevalence of PRISm has been studied in the general population and in smokers. According to COPDGene's earlier cross-sectional estimates, the global prevalence of PRISm is approximately 6.6%–17.6% [5, 23, 24]. A cohort study in the UK included 351,874 participants from the UK Biobank and found that 11% of the participants had PRISm at baseline [3], within the range of incidence predicted by the COPDGene study. Data from Schwartz et al. [6] showed that in their spirometry database at the academic medical center, the incidence of PRISm was approximately 17–24%, slightly higher than that reported in previous studies [6]. These studies suggested that PRISm is not uncommon. However, current studies on PRISm are most focused on the analysis of its epidemiology, clinical characteristics, and risk factors and the pathophysiological mechanisms are being few investigated.

In our previous study, we found that the airway wall area and emphysema index were not different between PRISm patients and chronic bronchitis patients without abnormal spirometry, whereas the PRISm patients had symptoms like GOLD 1 and 2 patients [25]. The PRISm group showed decreased lung capacity and higher mean lung density [25]. Kirby et al. found small airway pathological changes in early/mild COPD patients using CT total airway count, but their lung function parameters (such as FEV1 and FVC) are still in the normal range, suggesting that small airway impairment is appeared before lung function damage [26]. Furthermore, COPDGene study demonstrated that radiographic differences, such as decreased measurements of emphysema, gas trapping, total lung capacity, and segmental wall area thickness, were the robustly identified predictors of PRISm relative to COPD subjects [5]. These results indicated that the transition of PRISm to COPD might be related to impaired small airway function.

Our study focused on the changes in the pulmonary function of CB patients. Compared with the control group, the CB group with PRISm had lower predicted MEF25%, MEF50%, and MMEF25–75% levels. By contrast, the levels of R5-R20, Fres, and Ax increased. This showed that PRISm in CB patients can worsen small airway function, although the FEV1/FVC and FEV1/FEV6 ratio was considered normal in these patients. Furthermore, the PRISm group exhibited higher RV/TLC levels, indicating more serious air trapping than the control group. These results suggested PRISm patients have initiated the impairment of small airway before its progression to COPD. This result was in accordance with previous studies.

Small airway disease is reported to be a pathological feature in mild and moderate COPD [27]. However, there is still a lack of effective methods for identifying abnormal small airway function. The IOS is a noninvasive method to measure respiratory system resistance and reactance during normal breathing [14]. The IOS may be more sensitive in detecting small airway dysfunction and seems to be better correlated with small airway structures [17, 28]. Piortuneks' study concluded that it was appropriate to utilize the IOS to estimate respiratory dysfunction, especially in cases with small airways [29]. Additionally, our previously published research reported that IOS results had a good correlation with spirometry results [30]. The relationship between these two methods of pulmonary function testing is again confirmed in the present study. A high sensitivity of *R*5–*R*20, *X*5, and Ax, reflected in the predicted MMEF25–75% changes, makes the IOS particularly useful for the detection of mild lung injury.

Other studies have shown that a decline in FEV1 is a marker of COPD progression [31, 32]. In our study, the predicted FEV1% and RV/TLC values in the PRISm and COPD groups were similar, but both groups significantly differed from the control group. This implies that the degree of airflow obstruction and air trapping in PRISm patients is closer to that of COPD patients. However, there has been no evidence or guideline available to treat this condition earlier through medication [8].

In this study, subgroup analyses found that the levels of the predicted MEF25%, MEF50%, MMEF25–75%, *R*5–*R*20, and *X*5, and Ax did not significantly differ between the PRISm and GOLD grade 1 groups. Thus, we presumed that the severity of small airway dysfunction in patients with PRISm is similar to that of GOLD grade 1 patients. However, the sample size of our study is limited and follow-up of the PRISm group was not conducted. Moreover, this was a retrospective study. Thus, the presence of confounding factors is inevitable, and the current findings must be further investigated to provide more reliable evidence.

However, there are inevitable limitations in our study. First, this is a cross-sectional study and the longitudinal data were not included. The data on the exacerbations in patients with PRISm and COPD the year before the study are not available. Second, the FEV6 data were not complete. Third, this study only included the hospitalized patients and difference between hospitalized and nonhospitalized patients was not accessed. Therefore, further researches are still warrant to investigate.

## 7. Conclusions

In summary, not all patients with CB will progress to COPD. Earlier research demonstrated that many smokers without COPD had obvious respiratory disease [33] and that patients with respiratory symptoms, genetic risk factors, and a history of smoking are more likely to suffer from COPD [5, 34]. Thus, we were interested in methods to screen for lung function in these high-risk patients. Our findings showed that small airway dysfunction had already occurred in CB patients with PRISm, and we further confirmed the clinical value of the IOS, especially in assessing small airway function. Treatment strategies such as changes in lifestyle and medications should focus on early intervention regarding possible risk factors to reduce the progression of pulmonary function deterioration.

## Figures and Tables

**Table 1:** Baseline characteristics of patients in this study (Mean ± SD).

Variables	Control (*n* = 77)	PRISm (*n* = 80)	COPD (Gold 1, 2) (*n* = 186)	*P* value
Age (y)	67.42 ± 9.72	70.68 ± 10.8	68.17 ± 10.85	0.116
Sex (F/M)	20/57	19/61	26/160	
BMI (kg/m^2^)	24.03 ± 2.98	24.02 ± 4.03	23.77 ± 3.53	0.797
Smoking (pack-year)	31.86 ± 21.66	31.27 ± 18.18^‡‡^	41.9 ± 25.64^*∗*^	0.007
CAT	15.45 ± 8.82	16.25 ± 9.12^‡^	18.53 ± 8.25^*∗∗*^	0.014
mMRC	0.96 ± 0.98	1.43 ± 1.13^*∗∗*^	1.26 ± 1.1^*∗*^	0.024
WBC (×10^9^/L)	6.29 ± 2.33	6.38 ± 1.94^‡^	7.26 ± 3.28^*∗*^	0.011
*N* (%)	64.31 ± 11.10	65.47 ± 11.67^‡^	68.61 ± 11.41^*∗∗*^	0.010
*E* (%)	2.62 ± 2.27	3.08 ± 3.39	2.69 ± 2.7	0.504

BMI = body mass index, CAT = COPD Assessment Test score, mMRC = Modified British Medical Research Council, WBC = white blood cell count, *N* = neutrophil count, *E* = eosinophils count. ^*∗*^*p* < 0.05, ^*∗∗*^*p* < 0.01, versus Control, ^‡^*p* < 0.05, ^‡‡^*p* < 0.01, versus COPD.

**Table 2:** Analysis of variances results for three groups in this study (mean ± SD).

	Control (*n* = 77)	PRISm (*n* = 80)	COPD (*n* = 186)	*P* value
FEV1% pred	93 ± 10.71	63.96 ± 12.64^*∗∗*^	63.95 ± 11.44^*∗∗*^	<0.001
FVC% pred	92.26 ± 11.88	63.82 ± 13.53^*∗∗*^^,^^‡‡^	84.1 ± 13.92^*∗∗*^	<0.001
FEV1/FVC (%)	79.52 ± 6.54	78.46 ± 6.39^‡‡^	58.77 ± 7.12^*∗∗*^	<0.001
FEV1/FEV6 (%) ^#^	74.35 ± 10.32	72.75 ± 9.02^‡‡^	58.89 ± 7.46^*∗∗*^	<0.001
MEF25% pred	70.43 ± 31.87	47.07 ± 22.48^*∗∗*^^,^^‡‡^	31.04 ± 11.30^*∗∗*^	<0.001
MEF50% pred	75.13 ± 26.12	45.17 ± 15.88^*∗∗*^^,^^‡‡^	27.70 ± 8.66^*∗∗*^	<0.001
MMEF25–75% pred	73.46 ± 23.28	45.59 ± 15.01^*∗∗*^^,^^‡‡^	28.73 ± 8.64^*∗∗*^	<0.001
RV/TLC (%)	45.03 ± 7.05	53.92 ± 9.98^*∗∗*^	51.99 ± 9.45^*∗∗*^	<0.001
*R*5 (kPa s L^−1^)	0.41 ± 0.11	0.45 ± 0.11	0.48 ± 0.14^*∗∗*^	<0.001
*R*20 (kPa s L^−1^)	0.34 ± 0.08	0.34 ± 0.07	0.34 ± 0.07	0.93
*R*5–*R*20 (kPa s L^−1^)	0.07 ± 0.05	0.11 ± 0.07^*∗*^^,^^‡^	0.14 ± 0.09^*∗∗*^	<0.001
*X*5 (kPa s L^−1^)	−0.11 ± 0.05	−0.16 ± 0.08^*∗*^^,^^‡^	−0.19 ± 0.12^*∗∗*^	<0.001
Fres (1/s)	15.48 ± 5.41	18.04 ± 3.60^*∗*^^,^^‡^	19.85 ± 4.62^*∗∗*^	<0.001
Ax (kPa/L)	0.54 ± 0.49	0.97 ± 0.71^*∗*^^,^^‡‡^	1.46 ± 1.12^*∗∗*^	<0.001

^*∗*^ *p* < 0.05, ^*∗∗*^*p* < 0.01, versus control, ^‡^*p* < 0.05, ^‡‡^*p* < 0.01, versus COPD, ^#^ The number of patients for this parameter is control (*n* = 36), PRISm (*n* = 36), and COPD (*n* = 147) due to incomplete data of FEV6.

## Data Availability

The data used to support the findings of this study are available from the corresponding author upon request.

## Abbreviations

ANOVA: One-way analysis of variance
Ax: Area of reactance
CB: Chronic bronchitis
COPD: Chronic obstructive pulmonary disease
FEV1: Forced expiratory volume in 1 second
FVC: Forced vital capacity
IOS: Impulse oscillometry system
MEF: Maximal expiratory flow
MMEF: Maximal midexpiratory flow
PRISm: Preserved ratio impaired spirometry
SD: Standard deviation.
